# Analysis of dietary inflammatory potential and mortality in cancer survivors using NHANES data

**DOI:** 10.3389/fnut.2024.1467259

**Published:** 2024-09-13

**Authors:** Yemei Wu, Jing Yi, Qu Zhang

**Affiliations:** ^1^Hubei Cancer Hospital, Tongji Medical College, Huazhong University of Science and Technology, Wuhan, China; ^2^School of Public Health, Tongji Medical College, Huazhong University of Science and Technology, Wuhan, China; ^3^Department of Radiotherapy Center, Hubei Cancer Hospital, Tongji Medical College, Huazhong University of Science and Technology, Wuhan, China

**Keywords:** cancer survivors, dietary interventions, dietary inflammatory index, mortality, inflammation

## Abstract

**Background:**

In the United States, cancer is a leading cause of mortality, with inflammation playing a crucial role in cancer progression and prognosis. Diet, with its capacity to modulate inflammatory responses, represents a potentially modifiable risk factor in cancer outcomes.

**Methods:**

This study utilized data from the National Health and Nutrition Examination Survey (NHANES, 1999–2018) to investigate the association between the Dietary Inflammatory Index (DII), which reflects dietary-induced inflammation, and mortality among cancer survivors. A total of 3,011 participants diagnosed with cancer were included, with DII scores derived from dietary recall data. All-cause and cancer-related mortalities served as primary endpoints.

**Results:**

The study identified a significant linear positive correlation between higher DII scores and all-cause mortality among cancer survivors. Each unit increase in DII was associated with a 10% higher risk of all-cause mortality (hazard ratio [HR] per 1-unit increase, 1.10; 95% confidence interval [CI], 1.04–1.15). Similarly, a unit increase in DII was associated with a 13% higher risk of cancer-related mortality (HR per 1-unit increase, 1.13; 95% CI, 1.02–1.25). Kaplan–Meier analyses demonstrated higher all-cause mortality rates in individuals with elevated DII scores. Sensitivity analyses confirmed the robustness of these findings.

**Conclusion:**

Adoption of an anti-inflammatory diet, characterized by lower DII scores, may improve survival outcomes in cancer survivors. These results emphasize the critical role of dietary interventions in post-cancer care.

## Introduction

In the United States, cancer ranks as the second leading cause of mortality, with an estimated 1,958,310 new cases and 609,820 deaths expected in 2023 (1). The process of cancer initiation and progression is significantly influenced by inflammation, with elevated inflammation levels being associated with poor cancer prognosis (2). Dietary factors can influence cancer risk through various mechanisms, including modulation of the gut microbiome, reductions in oxidative stress, and maintenance of energy balance (3, 4).

The inflammatory potential of individual dietary components and dietary patterns is central to these mechanisms (5, 6). For instance, specific dietary elements such as ginger, garlic, and flaxseed have been demonstrated to reduce systemic inflammation by lowering markers such as C-reactive protein (CRP), interleukin-6 (IL-6), and tumor necrosis factor-alpha (TNF-α) (7). Additionally, following the Mediterranean diet correlates with decreased levels of systemic inflammatory markers like CRP and IL-6 (7, 8). Furthermore, compounds found in certain foods, such as omega-3 fatty acids (9) and polyphenols (10), exhibit anti-inflammatory properties. Therefore, dietary interventions may influence cancer prognosis by altering the body’s inflammatory status.

The Dietary Inflammatory Index (DII) is a data-driven instrument designed to assess the inflammatory potential of individual dietary intake. Through a comprehensive review of 1,943 articles and dietary databases from 11 countries, the DII encompasses 45 dietary parameters closely associated with 6 key inflammatory biomarkers (IL-1β, IL-4, IL-6, IL-10, TNF-α, and CRP). These parameters include essential nutrients and bioactive compounds such as fatty acids, antioxidants, vitamins, minerals, dietary fiber, and flavonoids (11). These components influence the inflammatory process through various mechanisms. For instance, saturated and polyunsaturated fatty acids (particularly omega-3 and omega-6 fatty acids) in dietary fat can modulate cell membrane fluidity and signal transduction, directly impacting the regulation of inflammatory responses (12–14). Additionally, antioxidants like vitamins C, E, and carotenoids mitigate oxidative stress by neutralizing free radicals, thereby reducing inflammation intensity (15, 16). Vitamins such as A, B-complex, C, D, E, and K play crucial roles in multiple immunoregulatory pathways, with vitamin D being particularly notable for its regulation of chronic inflammation (17, 18). Minerals including calcium, magnesium, zinc, iron, and selenium influence inflammatory states through various metabolic pathways (19, 20). Dietary fiber modulates the inflammatory response by regulating gut microbiota and promoting the production of short-chain fatty acids (21, 22). Moreover, phytochemicals like flavonoids, recognized for their significant anti-inflammatory, antioxidant, and immunomodulatory properties, further enhance the comprehensiveness of the DII as a tool for assessing the inflammatory potential of a diet (23, 24). Each component is assigned a value based on its effect on these markers, yielding an overall score indicative of the diet’s inflammatory potential. Positive DII scores signify pro-inflammatory diets, and negative scores denote anti-inflammatory effects (25).

The DII is designed to capture and quantify the cumulative effects of various nutrients and bioactive compounds on the inflammatory response. It provides a standardized scoring system that effectively evaluates the overall impact of individual or population dietary patterns on chronic inflammation. This tool is valuable not only for investigating the relationship between diet and inflammation but also for facilitating comparisons across different studies in large-scale epidemiological research, thereby supporting more precise and reliable scientific conclusions.

Higher DII scores may increase mortality risk in some cancer survivors, particularly those who were diagnosed with colorectal or breast cancer (26–30). Similar findings have been observed in specific populations of cancer survivors. For instance, a nationwide prospective cohort study in the United States involving postmenopausal women found that adopting an anti-inflammatory diet after being diagnosed with primary invasive cancer could improve survival rates (31). However, existing research has primarily focused on specific cancer types or particular populations, such as patients with colorectal cancer, breast cancer, or postmenopausal women. Research examining the association between dietary inflammatory potential and survival outcomes in the overall population of cancer survivors is notably scarce.

This study addresses this gap by exploring the impact of dietary inflammatory potential on survival outcomes among the overall population of cancer survivors. Specifically, we aimed to investigate the association between dietary inflammatory potential and post-diagnosis mortality rates in patients with cancer, including all-cause mortality and cancer-specific mortality, using a large and comprehensive database to enhance the reliability of our findings.

## Materials and methods

### Study population

This study leveraged datasets from the National Health and Nutrition Examination Survey (NHANES), a program executed by the National Center for Health Statistics, which is part of the Centers for Disease Control and Prevention (CDC). NHANES is a nationally representative cross-sectional survey assessing the health status of Americans (32) and has been conducted continuously since 1999, with data released biennially. Health and nutrition data are collected through a multistage, stratified, and clustered sampling method, which includes interviews, physical examinations, and laboratory tests. NHANES is currently the only nationwide survey at the national level that provides comprehensive data on nutrient intake from foods, beverages, and dietary supplements across all age groups in the United States. Detailed information regarding NHANES is available elsewhere (33).

**Figure 1:**
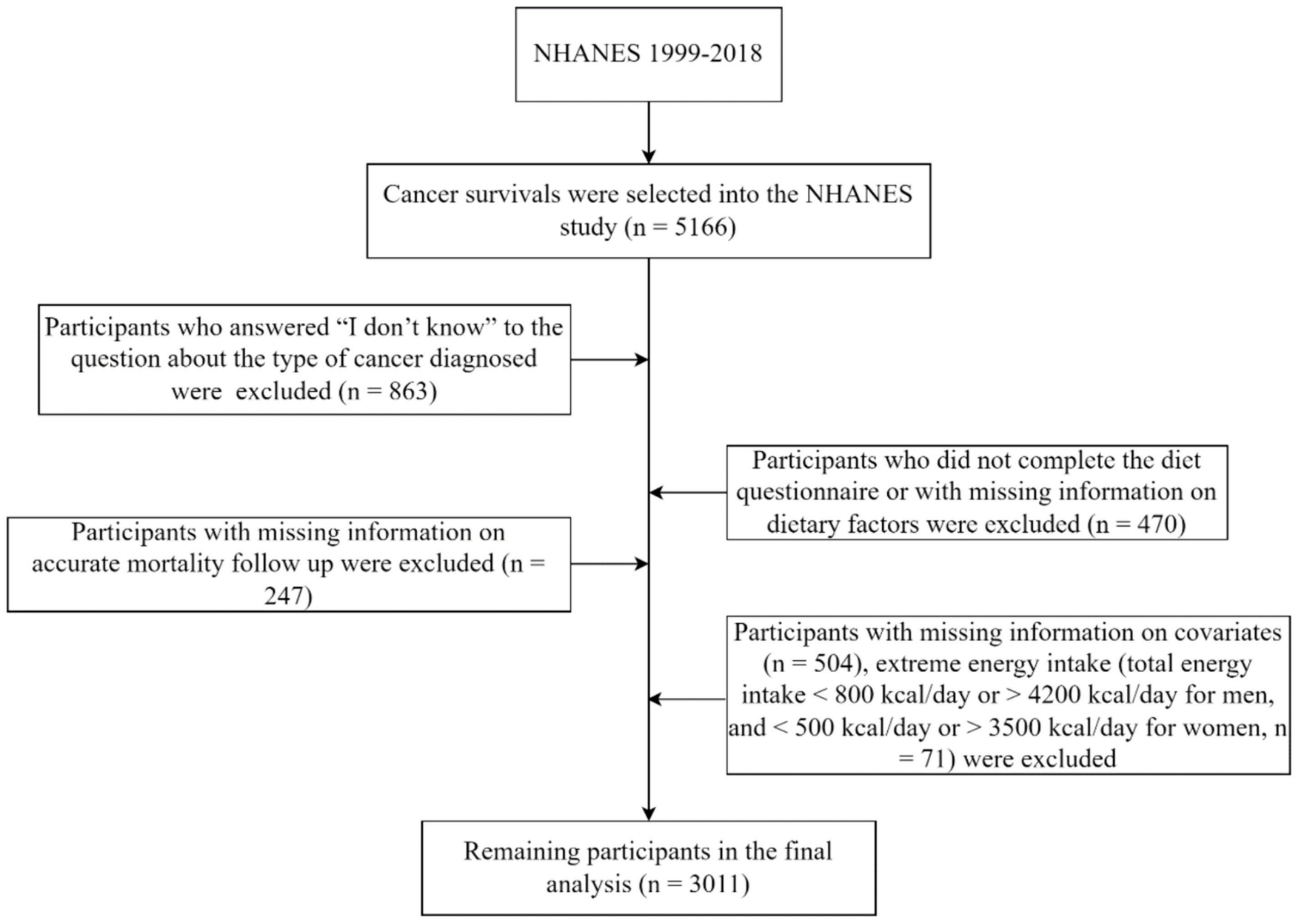
The flow chart of selecting a full analysis set.

This study included 5,166 participants aged ≥18 years who were diagnosed with cancer, based on data from NHANES (1999–2018). A cancer diagnosis was determined by participants answering “yes” to the interview question, “Were you ever told by a doctor or other health professional that you had cancer or a malignancy of any kind?.” We excluded participants who responded “I do not know” to the type of cancer diagnosed (*n* = 863); those who did not complete the dietary questionnaire or had missing dietary information (*n* = 470); those with missing covariate information (*n* = 504); those lacking accurate mortality follow-up information (*n* = 247); and those with abnormal daily caloric consumption, including males with intakes <800 or > 4,200 kcal/day and females with intakes <500 or > 3,500 kcal/day (*n* = 71). Ultimately, the analysis encompassed the data from 3,011 participants (Figure 1).

### Dietary assessment

NHANES collected 24 h dietary recall data from participants using professional interviewers. Between 1999 and 2002, these interviews were conducted once at mobile examination centers; during 2003–2018, a second interview was conducted via telephone approximately 3–10 days later. When second recall data were available, the average food intake over the two 24 h periods was calculated. This method has been validated and found to be more accurate than other approaches (34). The aggregate caloric and nutritional uptakes were calculated using the dietary intake data of the enrolled participants, employing the United States Department of Agriculture Food and Nutrient Database for Dietary Studies as the analytical tool (35, 36).

### DII calculation

Individual DII scores were calculated using participant dietary intake data acquired through dietary surveys (Supplementary Table S1). Elevated DII scores are indicative of a diet rich in pro-inflammatory elements, and reduced scores reflect a diet predominantly composed of anti-inflammatory constituents. Supplementary Figure S1 provides detailed information on the steps involved in determining DII scores. Participants were divided into tertiles based on their DII scores. Supplementary Table S2 presents the nutrient composition and intake levels corresponding to each DII tertile.

### Covariate assessment

Covariate information was obtained from baseline questionnaires, encompassing variables such as age, sex, race/ethnicity, level of education, household economic status, marital status, history of smoking, duration from cancer diagnosis to baseline, body mass index (BMI), energy intake, and comorbidity history. Participants designated as “non-smokers” reported a lifetime cigarette consumption that did not exceed 100. The household income-to-poverty ratio (PIR) was utilized to stratify household income. Comorbidities included diagnoses of diabetes, hyperlipidemia, and cardiovascular disease.

### Outcome assessment

The primary endpoints of our study were all-cause and cancer-related mortality. We used the National Death Index to record mortality; the National Center for Health Statistics provides further details on the matching technique implemented to obtain these data (37). The 10th edition of the International Classification of Diseases (ICD-10) was used to categorize causes of death, with codes C00–C97 indicating cancer-related mortality.

### Statistical analyses

All analyses accounted for the sample weights derived from the intricate sampling framework of NHANES. To evaluate differences in baseline characteristics, analysis of variance was conducted on continuous data, whereas categorical data were assessed using the chi-square test. Participants were classified into three DII tertiles, with the initial tertile designated as the comparative benchmark.

The correlation between DII and mortality from all causes and cancer was evaluated with hazard ratios (HRs) and 95% confidence intervals (CIs), generated from Cox proportional hazards regression analysis. Two models were developed to correct for confounders. Model 1 incorporated adjustments for age, sex, energy intake tertiles, and time from cancer diagnosis to baseline. Model 2 expanded these adjustments to encompass comorbidity count, educational attainment, marital status, PIR, race/ethnicity, smoking habits, and BMI. Given that alcohol intake was inherently incorporated into the DII computation, it was excluded from the models. Trend analyses were executed by treating the median intake values of categorical variables as a continuous variable. Furthermore, continuous DII values were utilized to calculate risk estimates corresponding to each 1-unit increment. The analysis employed multivariate restricted cubic spline to scrutinize the dose–response correlation between DII and mortality. Kaplan–Meier curves were used to depict mortality across the DII tertiles.

Stratified analyses were performed based on age, sex, lifestyle factors (smoking status, BMI <25 or ≥ 25 kg/m^2^), comorbidity count (0 or ≥ 1), and follow-up duration (≤15 or > 15 person-years). Log-likelihood tests compared models with and without continuous DII and interaction terms to assess effect modification.

Sensitivity analyses were also performed. First, individuals diagnosed with cancer less than a year before baseline were excluded to account for dietary changes due to adjuvant therapy (*n* = 2,802). Then, to reduce potential overadjustment bias, all variables except BMI were adjusted, as BMI could mediate the relationship between DII and mortality (*n* = 3,011). Statistical analyses were conducted using SAS 9.4 (Cary, NC, United States) and R 4.1.3 (Vienna, Austria), with significance set at a *p* value <0.05, adopting a two-tailed test approach.

## Results

### Participant characteristics

This study included a final cohort of 3,011 participants (mean age, 62.66 years; 44.24% male). Participants were stratified into tertiles based on their DII scores: 1,004 participants in the high DII group (T3, representing the most pro-inflammatory diet), 1,004 in the medium DII group (T2), and 1,003 in the low DII group (T1, representing the most anti-inflammatory diet). The range of DII scores was from −4.54 to 4.93. According to the baseline characteristics presented in Table 1, the T3 group predominantly consisted of younger, well-educated females who were current smokers. In comparison with the T1 group, individuals in the higher DII category were less likely to be married or of white ethnicity and reported lower energy intake. Additionally, those in the high DII group had higher rates of obesity, more comorbidities, and lower household incomes.

**Table 1:** Baseline characteristics of participants from the US National Health and Nutrition Examination Survey (NHANES) according to tertiles of the dietary inflammatory index (DII) (*n* = 3,011)^a^.

Characteristics	All (*n* = 3,011)	Tertile 1 (−4.58–−0.94) *n* = 1,003	Tertile 2 (−0.94–0.88) *n* = 1,004	Tertile 3 (0.88–4.93) *n* = 1,004	*p* value
Age (y), mean (SE)	62.66 (0.44)	64.07 (0.57)	63.16 (0.70)	60.37 (0.76)	0.0002
PIR, mean (SE)	3.24 (0.05)	3.64 (0.07)	3.33 (0.09)	2.64 (0.08)	<0.0001
Years from cancer diagnosis to baseline, mean (SE)	11.12 (0.30)	10.86 (0.34)	11.43 (0.55)	11.10 (0.55)	0.6604
Energy intake (kcal/day), mean (SE)	1928.75 (16.19)	2301.01 (23.46)	1902.33 (24.31)	1503.85 (20.15)	<0.0001
Sex, Male (*n*, %)	1,463 (44.24)	612 (56.50)	490 (43.46)	361 (30.14)	<0.0001
Smoking status (*n*, %)					<0.0001
Never	1,289 (43.13)	443 (45.94)	450 (45.22)	396 (37.35)	
Former	1,292 (40.16)	477 (46.01)	424 (38.71)	391 (34.65)	
Current	430 (16.71)	83 (8.05)	130 (16.07)	217 (28.00)	
Marital status, Married (*n*, %)	1896 (67.15)	680 (70.37)	637 (69.08)	579 (61.04)	0.0047
Educational level (*n*, %)					<0.0001
College or above	659 (14.37)	135 (9.88)	206 (12.29)	318 (22.20)	
High school or equivalent	705 (22.18)	209 (17.65)	228 (22.17)	268 (27.71)	
Less than high school	1,647 (63.45)	659 (72.47)	570 (65.54)	418 (50.09)	
Race, White (*n*, %)	2,240 (88.97)	806 (91.94)	746 (88.78)	688 (85.55)	<0.0001
BMI group (kg/m^2^) (*n*, %)					0.0325
<18.5	53 (1.91)	15 (1.56)	15 (1.56)	23 (2.72)	
18.5–24.9	831 (29.36)	315 (32.99)	255 (24.44)	261 (30.45)	
25.0–29.9	1,090 (35.22)	385 (34.93)	372 (38.33)	333 (32.09)	
≥30.0	1,037 (33.51)	288 (30.52)	362 (35.67)	387 (34.73)	
History of comorbidities, yes (*n*, %)	1957 (58.81)	612 (54.76)	667 (63.06)	678 (58.99)	0.0244

a All estimates accounted for complex survey designs in NHANES. Values were mean ± standard error for continuous variables and numbers (percentages) for categorical variables. Abbreviation and acronyms: PIR family income-poverty ratio; BMI body mass index.

### All-cause and cancer-related mortality

Over a median follow-up of 11.25 years, there were 1,193 (41.07%) deaths, of which 388 (12.89%) were attributed to cancer.

The results of the Cox regression models are detailed in Table 2. Both models indicated a significant positive association between DII tertiles and all-cause mortality among patients with cancer. Comparing the highest and lowest tertiles, Model 1 yielded an HR of 1.31 (95% CI, 1.05–1.64; P for trend = 0.02), closely aligning with that of Model 2 (HR, 1.34; 95% CI, 1.07–1.69; P for trend = 0.01). Additionally, when DII was analyzed as a continuous variable, the harmful impact of a pro-inflammatory diet was evident, with an HR of 1.10 per 1-unit increase (95% CI, 1.04–1.15).

**Table 2:** Associations of the dietary inflammatory index (DII) with all-cause and cancer mortality among cancer population in the US National Health and Nutrition Examination Survey (NHANES)^a^.

Tertile of DII	Death from any cause (*n*)	Person-years	Hazard ratio (95% confidence interval)	
			Model 1^b^	Model 2^c^
All-cause mortality				
Tertile 1	346	8,907	1.00 (reference)	1.00 (reference)
Tertile 2	416	8,870	1.27 (1.03–1.57)	1.17 (0.95–1.45)
Tertile 3	431	8,807	1.68 (1.36–2.07)	1.31 (1.05–1.64)
P trend			<0.0001	0.0213
Per 1-unit DII increment	1,193	26,584	1.16 (1.1–1.22)	1.10 (1.04–1.15)
Cancer mortality				
Tertile 1	117	8,907	1.00 (reference)	1.00 (reference)
Tertile 2	124	8,870	1.32 (0.89–1.97)	1.27 (0.86–1.86)
Tertile 3	147	8,807	1.68 (1.12–2.51)	1.31 (0.86–1.98)
P trend			0.01	0.19
Per 1-unit DII increment	388	26,584	1.19 (1.06–1.32)	1.13 (1.02–1.25)

a All estimates accounted for complex survey designs in NHANES.

b Model 1: adjusted for age, sex, tertiles of energy intake and years from cancer diagnosis to baseline.

c Model 2: adjusted for all variables in model 1 and further for PIR, marital status, educational level, race/ethnicity, baseline BMI group, smoking status, and history of comorbidities.

With regard to cancer-related mortality, Model 1 showed a significant association with DII (HR for T3 vs. T1, 1.68; 95% CI, 1.12–2.51; P for trend = 0.01). However, this association was not observed with Model 2 (HR for T3 vs. T1, 1.31; 95% CI, 0.86–1.98; P for trend = 0.19). When considering DII as a continuous variable, Model 2 did reveal a significant detrimental effect (HR per 1-unit increase, 1.13; 95% CI, 1.02–1.25).

Restricted cubic spline plots (Supplementary Figure S2) indicated a linear increase in both all-cause and cancer-related mortality with increasing DII (P for linearity <0.05; P for non-linearity >0.05).

**Figure 2:**
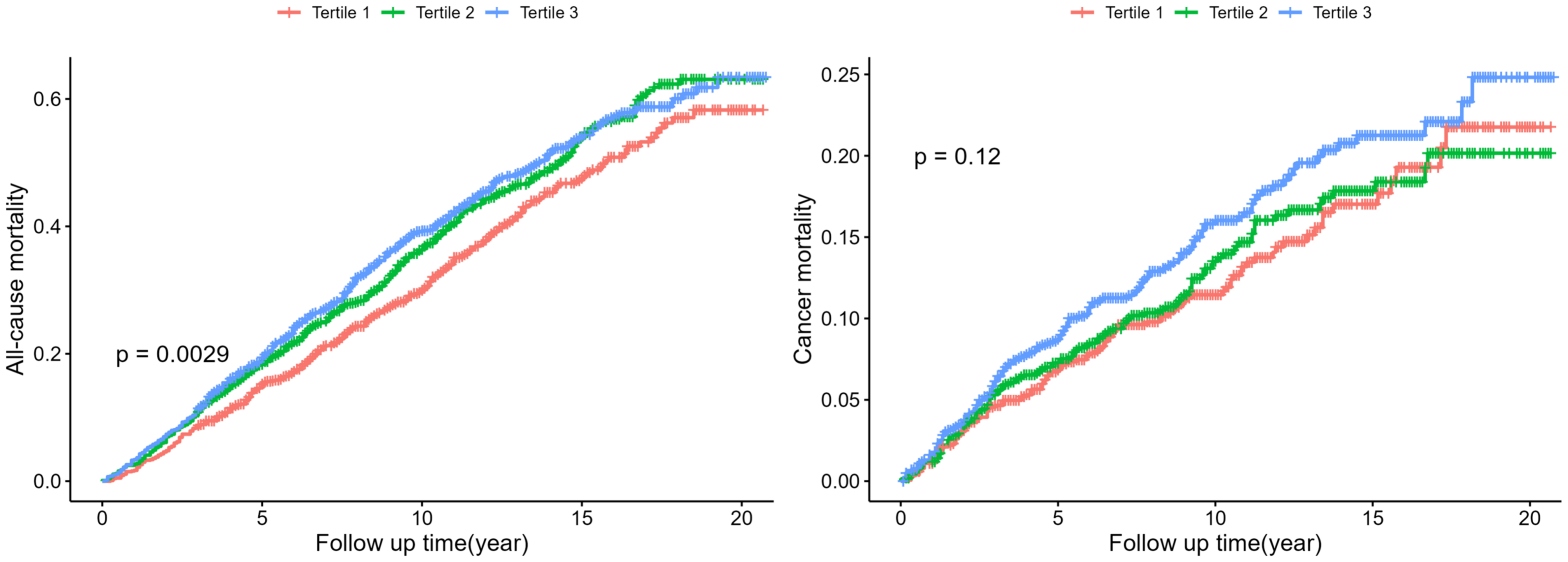
Kaplan Meier plots for all-cause mortality and cancer mortality by tertiles of the DII. Log-Rank-Test was used to evaluate differences.

Kaplan–Meier plots (Figure 2) suggested a higher all-cause mortality for participants with higher DII than for those with lower DII (*p* = 0.0029), although no significant difference was observed in cancer-related mortality (*p* = 0.12). Stratified analyses did not reveal any significant interaction between DII and mortality (all interaction *p* values >0.05; Supplementary Figure S3).

Sensitivity analyses demonstrated that the results remained consistent after excluding participants diagnosed with cancer within 1 year from baseline and after adjusting for all variables except BMI (Supplementary Table S3).

## Discussion

In this nationally representative study, a linear association was identified between the DII scores post-cancer diagnosis and the risk of mortality, encompassing both all-cause and cancer-related fatalities. Specifically, each unit increase in DII corresponded with a 10% increase in all-cause mortality risk (95% CI, 4–15%) and a 13% increase in cancer-related mortality risk (95% CI, 2–25%). Based on our current understanding, this research represents a pioneering effort to explore the correlation between DII and all-cause and cancer-related mortality within the population of cancer survivors.

Our investigation revealed a significant positive correlation between DII and all-cause mortality in cancer survivors, and the Kaplan–Meier curve and sensitivity analyses yielded similar results. These findings are consistent with those of a previous sub-analysis of the Iowa Women’s Health Study which investigated the correlation between dietary inflammatory potential and mortality in older female cancer survivors, revealing that an anti-inflammatory diet and supplements could improve survival rates in postmenopausal cancer survivors (31). Additionally, substantial evidence links the Mediterranean diet and higher Healthy Eating Index (HEI) scores to improved cancer survival rates due to their anti-inflammatory properties (38–40). Furthermore, healthy dietary behaviors are associated with reduced all-cause mortality risk, largely due to the intake of anti-inflammatory compounds found in vegetables, fruits, whole grains, and legumes (41–43). In contrast, prospective studies and meta-analyses suggest that diets with a high inflammatory potential are linked to an elevated cancer incidence (44–46).

Contrary to our findings regarding all-cause mortality, our study did not reveal a statistically significant correlation between DII and cancer-related mortality among cancer survivors. Previous research indicates that an anti-inflammatory diet may reduce mortality in survivors of specific cancers such as colorectal, breast, and prostate cancers (28–30, 47). Although these findings support the protective role of an anti-inflammatory diet in reducing mortality in certain cancer survivors, they are inconsistent with our results. However, our analysis should be interpreted cautiously because detailed data on cancer treatment regimens, staging, grading, and specific causes were lacking.

The mechanisms connecting dietary inflammatory potential to cancer-related mortality are not well understood, though several plausible pathways have been proposed. A diet with high inflammatory potential can upregulate inflammatory factors (8), promoting cancer cell proliferation, survival, and migration, thereby increasing the risk of cancer-related death (48, 49). This diet may also accelerate telomere shortening, which is linked to higher all-cause mortality risk (50, 51). Moreover, it is associated with elevated concentrations of very low-density lipoprotein, low-density lipoprotein, and TNF-α, all of which correlate with higher mortality risk (52–54). Saturated fats, prevalent in pro-inflammatory diets, are connected to increased risks of all-cause mortality, cancer, and cardiovascular disease deaths (55). Given the critical role of inflammation in tumor progression, dietary factors likely influence disease susceptibility and cancer risk by affecting inflammatory pathways (26, 56, 57), supporting the observed associations.

This study has several strengths. First, we utilized a large, nationally representative sample and adjusted for covariates to ensure the robustness and generalizability of our findings. Further validation of our results was achieved through sensitivity analyses, underscoring the reliability of our conclusions. Moreover, the application of DII in our study was pivotal, given its specialized role in quantifying the overall inflammatory impact of dietary intake. Unlike other dietary scoring systems (e.g., HEI, Mediterranean Diet Score), DII provides standardized quantification, allowing for consistent comparisons across different studies (58–60). By analyzing dietary patterns and food groups rather than individual nutrients, we captured the combined effects of various dietary components, providing a comprehensive view of individual dietary habits and supporting reliable conclusions and precise statistical outcomes (61).

However, the limitations of this study should be acknowledged. First, NHANES is a cross-sectional survey, which precludes establishing a causal relationship between DII and mortality among cancer survivors. Future research will be required to better define this relationship. Second, while the reliance on one or two 24 h dietary recalls per participant may not fully capture long-term dietary habits, studies have shown that this method remains an effective means of reasonably estimating overall dietary intake in population studies (62, 63). Following input and validation/cross-validation, expert panels reached a consensus in multiple workshops, agreeing that this approach is appropriate for large-scale surveys (35). Third, despite the inherent subjective bias in self-reported dietary information, the robustness of our findings was assessed through sensitivity analyses, which demonstrated consistent results. Lastly, due to the lack of information on disease severity or treatment, we could not perform in-depth analyses on the associations between DII and prognosis among different groups based on cancer treatment regimens, staging, grading, and causes of cancer-related mortality, as well as their potential mechanisms.

## Conclusion

Compared with a pro-inflammatory diet, a diet rich in anti-inflammatory components, denoted by a diminished DII, was inversely associated with all-cause mortality among cancer survivors, although it did not significantly impact cancer-related mortality. These findings suggest that anti-inflammatory dietary patterns may offer survival benefits to cancer survivors. Large-scale future cohort studies or clinical trials are imperative to substantiate these results and investigate the potential influence of dietary-induced inflammation on survival outcomes via other clinical or biological mechanisms.

## Data Availability

The original contributions presented in the study are included in the article/Supplementary material, further inquiries can be directed to the corresponding author.

## References

[1] Siegel RL Miller KD Wagle NS Jemal A. Cancer statistics, 2023. CA Cancer J Clin. (2023) 73:17–48. doi: 10.3322/caac.21763, PMID: 36633525

[2] Greten FR Grivennikov SI. Inflammation and cancer: triggers, mechanisms, and consequences. Immunity. (2019) 51:27–41. doi: 10.1016/j.immuni.2019.06.025, PMID: 31315034 PMC6831096

[3] Cryan JF O'Riordan KJ Cowan CSM Sandhu KV Bastiaanssen TFS Boehme M. The microbiota-gut-brain Axis. Physiol Rev. (2019) 99:1877–2013. doi: 10.1152/physrev.00018.2018, PMID: 31460832

[4] Tosti V Bertozzi B Fontana L. Health benefits of the Mediterranean diet: metabolic and molecular mechanisms. J Gerontol A Biol Sci Med Sci. (2018) 73:318–26. doi: 10.1093/gerona/glx227 29244059 PMC7190876

[5] Di Giosia P Stamerra CA Giorgini P Jamialahamdi T Butler AE Sahebkar A. The role of nutrition in inflammaging. Ageing Res Rev. (2022) 77:101596. doi: 10.1016/j.arr.2022.101596, PMID: 35219904

[6] Zitvogel L Pietrocola F Kroemer G. Nutrition, inflammation and cancer. Nat Immunol. (2017) 18:843–50. doi: 10.1038/ni.3754, PMID: 28722707

[7] Schwingshackl L Hoffmann G. Mediterranean dietary pattern, inflammation and endothelial function: a systematic review and meta-analysis of intervention trials. Nutr Metab Cardiovasc Dis. (2014) 24:929–39. doi: 10.1016/j.numecd.2014.03.003, PMID: 24787907

[8] Shivappa N Steck SE Hurley TG Hussey JR Hébert JR. Designing and developing a literature-derived, population-based dietary inflammatory index. Public Health Nutr. (2014) 17:1689–96. doi: 10.1017/S1368980013002115, PMID: 23941862 PMC3925198

[9] Reinders I Virtanen JK Brouwer IA Tuomainen TP. Association of serum n-3 polyunsaturated fatty acids with C-reactive protein in men. Eur J Clin Nutr. (2012) 66:736–41. doi: 10.1038/ejcn.2011.195, PMID: 22113248

[10] Harms LM Scalbert A Zamora-Ros R Rinaldi S Jenab M Murphy N. Plasma polyphenols associated with lower high-sensitivity C-reactive protein concentrations: a cross-sectional study within the European prospective investigation into Cancer and nutrition (EPIC) cohort. Br J Nutr. (2020) 123:198–208. doi: 10.1017/S0007114519002538, PMID: 31583990 PMC7015881

[11] Hébert JR Shivappa N Wirth MD Hussey JR Hurley TG. Perspective: the dietary inflammatory index (DII)-lessons learned, improvements made, and future directions. Adv Nutr. (2019) 10:185–95. doi: 10.1093/advances/nmy071, PMID: 30615051 PMC6416047

[12] Djuricic I Calder PC. Beneficial outcomes of Omega-6 and Omega-3 polyunsaturated fatty acids on human health: An update for 2021. Nutrients. (2021) 13:2421. doi: 10.3390/nu13072421, PMID: 34371930 PMC8308533

[13] Fritsche KL. The science of fatty acids and inflammation. Adv Nutr. (2015) 6:293S–301S. doi: 10.3945/an.114.006940, PMID: 25979502 PMC4424767

[14] Mann ER Lam YK Uhlig HH. Short-chain fatty acids: linking diet, the microbiome and immunity. Nat Rev Immunol. (2024) 24:577–95. doi: 10.1038/s41577-024-01014-8, PMID: 38565643

[15] Hajizadeh-Sharafabad F Zahabi ES Malekahmadi M Zarrin R Alizadeh M. Carotenoids supplementation and inflammation: a systematic review and meta-analysis of randomized clinical trials. Crit Rev Food Sci Nutr. (2022) 62:8161–77. doi: 10.1080/10408398.2021.1925870, PMID: 33998846

[16] Rubin LP Ross AC Stephensen CB Bohn T Tanumihardjo SA. Metabolic effects of inflammation on vitamin a and carotenoids in humans and animal models. Adv Nutr. (2017) 8:197–212. doi: 10.3945/an.116.014167, PMID: 28298266 PMC5347109

[17] McKinley MC. Effect of vitamin D and Omega-3 supplements on systemic inflammation. Clin Chem. (2019) 65:1469–70. doi: 10.1373/clinchem.2019.312272, PMID: 31699703

[18] Rapa SF Di Iorio BR Campiglia P Heidland A Marzocco S. Inflammation and oxidative stress in chronic kidney disease-potential therapeutic role of minerals, vitamins and plant-derived metabolites. Int J Mol Sci. (2019) 21:263. doi: 10.3390/ijms21010263 31906008 PMC6981831

[19] Cheng X Wei Y Wang R Jia C Zhang Z An J. Associations of essential trace elements with epigenetic aging indicators and the potential mediating role of inflammation. Redox Biol. (2023) 67:102910. doi: 10.1016/j.redox.2023.102910, PMID: 37793240 PMC10562911

[20] González-Domínguez Á Domínguez-Riscart J Millán-Martínez M Mateos-Bernal RM Lechuga-Sancho AM González-Domínguez R. Trace elements as potential modulators of puberty-induced amelioration of oxidative stress and inflammation in childhood obesity. Biofactors. (2023) 49:820–30. doi: 10.1002/biof.1946, PMID: 36929162

[21] Shivakoti R Biggs ML Djoussé L Durda PJ Kizer JR Psaty B. Intake and sources of dietary Fiber, inflammation, and cardiovascular disease in older US adults. JAMA Netw Open. (2022) 5:e225012. doi: 10.1001/jamanetworkopen.2022.5012, PMID: 35357453 PMC8972036

[22] Ma W Nguyen LH Song M Wang DD Franzosa EA Cao Y. Dietary fiber intake, the gut microbiome, and chronic systemic inflammation. Gastroenterology. (2020) 158:S-200. doi: 10.1016/S0016-5085(20)31183-5, PMID: 34140026 PMC8212460

[23] González R Ballester I López-Posadas R Suárez MD Zarzuelo A Martínez-Augustin O. Effects of flavonoids and other polyphenols on inflammation. Crit Rev Food Sci Nutr. (2011) 51:331–62. doi: 10.1080/10408390903584094, PMID: 21432698

[24] Li G Ding K Qiao Y Zhang L Zheng L Pan T. Flavonoids regulate inflammation and oxidative stress in Cancer. Molecules. (2020) 25:5628. doi: 10.3390/molecules25235628, PMID: 33265939 PMC7729519

[25] Shivappa N Hebert JR Kivimaki M Akbaraly T. Alternative healthy eating index 2010, dietary inflammatory index and risk of mortality: results from the Whitehall II cohort study and meta-analysis of previous DII and mortality studies – CORRIGENDUM. Br J Nutr. (2017) 118:639. doi: 10.1017/S0007114517002719, PMID: 29056107

[26] Castro-Espin C Agudo A. The role of diet in prognosis among cancer survivors: a systematic review and meta-analysis of dietary patterns and diet interventions. Nutrients. (2022) 14:348. doi: 10.3390/nu14020348, PMID: 35057525 PMC8779048

[27] Park SH Hoang T Kim J. Dietary factors and breast cancer prognosis among breast cancer survivors: a systematic review and meta-analysis of cohort studies. Cancers (Basel). (2021) 13:5329. doi: 10.3390/cancers13215329, PMID: 34771493 PMC8582373

[28] Wang K Sun JZ Wu QX Li ZY Li DX Xiong YF. Long-term anti-inflammatory diet in relation to improved breast cancer prognosis: a prospective cohort study. NPJ Breast Cancer. (2020) 6:36. doi: 10.1038/s41523-020-00179-4, PMID: 32821804 PMC7426822

[29] Wesselink E Valk AW Kok DE Lanen AV de Wilt JH van Kouwenhoven EA. Postdiagnostic intake of a more proinflammatory diet is associated with a higher risk of recurrence and all-cause mortality in colorectal cancer survivors. Am J Clin Nutr. (2023) 117:243–51. doi: 10.1016/j.ajcnut.2022.11.018, PMID: 36811565

[30] Ratjen I Shivappa N Schafmayer C Burmeister G Nothlings U Hampe J. Association between the dietary inflammatory index and all-cause mortality in colorectal cancer long-term survivors. Int J Cancer. (2019) 144:1292–301. doi: 10.1002/ijc.31919, PMID: 30303515

[31] Zheng J Tabung FK Zhang J Caan B Hebert JR Kroenke CH. Association between dietary inflammatory potential and mortality after cancer diagnosis in the Women’s health initiative. Br J Cancer. (2023) 128:606–17. doi: 10.1038/s41416-022-02079-9, PMID: 36482189 PMC9938285

[32] National Center for Health Statistics. National Health and Nutrition Examination Survey (2023). Available at: https://wwwn.cdc.gov/nchs/nhanes/Default.aspx (Accessed October 1, 2023).

[33] Centers for Disease Control and Prevention (CDC). About the National Health and nutrition examination survey. (2023). Available at: https://www.cdc.gov/nchs/nhanes/about_nhanes.htm (Accessed October 1, 2023).

[34] Basiotis PP Welsh SO Cronin FJ Kelsay JL Mertz W. Number of days of food intake records required to estimate individual and group nutrient intakes with defined confidence. J Nutr. (1987) 117:1638–41. doi: 10.1093/jn/117.9.1638, PMID: 3655942

[35] Ahluwalia N Dwyer J Terry A Moshfegh A Johnson C. Update on NHANES dietary data: focus on collection, release, analytical considerations, and uses to inform public policy. Adv Nutr. (2016) 7:121–34. doi: 10.3945/an.115.009258, PMID: 26773020 PMC4717880

[36] Moshfegh AJ Rhodes DG Baer DJ Murayi T Clemens JC Rumpler WV. The US Department of Agriculture Automated Multiple-Pass Method reduces bias in the collection of energy intakes. Am J Clin Nutr. (2008) 88:324–32. doi: 10.1093/ajcn/88.2.324, PMID: 18689367

[37] National Center for Health Statistics. Public-use linked mortality files. (2019). Available at: https://www.cdc.gov/nchs/data-linkage/mortality-public.htm# (Accessed October 1, 2023).

[38] Di Maso M Augustin LSA Toffolutti F Stocco C Dal Maso L Jenkins DJA. Adherence to Mediterranean diet, physical activity and survival after prostate cancer diagnosis. Nutrients. (2021) 13:243. doi: 10.3390/nu13010243, PMID: 33467042 PMC7829941

[39] Castro-Espin C Bonet C Crous-Bou M Nadal-Zaragoza N Tjonneland A Mellemkjaer L. Association of Mediterranean diet with survival after breast cancer diagnosis in women from nine European countries: results from the EPIC cohort study. BMC Med. (2023) 21:225. doi: 10.1186/s12916-023-02934-3, PMID: 37365585 PMC10294413

[40] Shan Z Wang F Li Y Baden MY Bhupathiraju SN Wang DD. Healthy eating patterns and risk of total and cause-specific mortality. JAMA Intern Med. (2023) 183:142–53. doi: 10.1001/jamainternmed.2022.6117, PMID: 36622660 PMC9857813

[41] Inoue-Choi M Robien K Lazovich D. Adherence to the WCRF/AICR guidelines for cancer prevention is associated with lower mortality among older female cancer survivors. Cancer Epidemiol Biomarkers Prev. (2013) 22:792–802. doi: 10.1158/1055-9965.EPI-13-0054, PMID: 23462914 PMC3650116

[42] Song M Wu K Meyerhardt JA Ogino S Wang M Fuchs CS. Fiber intake and survival after colorectal cancer diagnosis. JAMA Oncol. (2018) 4:71–9. doi: 10.1001/jamaoncol.2017.3684, PMID: 29098294 PMC5776713

[43] Van Blarigan EL Fuchs CS Niedzwiecki D Zhang S Saltz LB Mayer RJ. Association of survival with adherence to the American Cancer Society nutrition and physical activity guidelines for cancer survivors after colon cancer diagnosis: the CALGB 89803/Alliance trial. JAMA Oncol. (2018) 4:783–90. doi: 10.1001/jamaoncol.2018.0126, PMID: 29710284 PMC6145685

[44] Ryu I Kwon M Sohn C Shivappa N Hebert JR Na W. The association between dietary inflammatory index (DII) and cancer risk in Korea: a prospective cohort study within the KoGES-HEXA study. Nutrients. (2019) 11:2560. doi: 10.3390/nu11112560, PMID: 31652856 PMC6893737

[45] Fowler ME Akinyemiju TF. Meta-analysis of the association between dietary inflammatory index (DII) and cancer outcomes. Int J Cancer. (2017) 141:2215–27. doi: 10.1002/ijc.30922, PMID: 28795402 PMC6056732

[46] Namazi N Larijani B Azadbakht L. Association between the dietary inflammatory index and the incidence of cancer: a systematic review and meta-analysis of prospective studies. Public Health. (2018) 164:148–56. doi: 10.1016/j.puhe.2018.04.015, PMID: 30321762

[47] Zucchetto A Gini A Shivappa N Hebert JR Stocco C Dal Maso L. Dietary inflammatory index and prostate cancer survival. Int J Cancer. (2016) 139:2398–404. doi: 10.1002/ijc.30208, PMID: 27242333 PMC5585016

[48] Mierke CT. The fundamental role of mechanical properties in the progression of cancer disease and inflammation. Rep Prog Phys. (2014) 77:076602. doi: 10.1088/0034-4885/77/7/076602, PMID: 25006689

[49] Dibaba DT Judd SE Gilchrist SC Cushman M Pisu M Safford M. Association between obesity and biomarkers of inflammation and metabolism with cancer mortality in a prospective cohort study. Metabolism. (2019) 94:69–76. doi: 10.1016/j.metabol.2019.01.007, PMID: 30802456 PMC7401298

[50] García-Calzón S Zalba G Ruiz-Canela M Shivappa N Hébert JR Martínez JA. Dietary inflammatory index and telomere length in subjects with a high cardiovascular disease risk from the PREDIMED-NAVARRA study: Cross-sectional and longitudinal analyses over 5 y. Am J Clin Nutr. (2015) 102:897–904. doi: 10.3945/ajcn.115.116863, PMID: 26354530 PMC4588745

[51] Wang Q Zhan Y Pedersen NL Fang F Hägg S. Telomere length and all-cause mortality: a meta-analysis. Ageing Res Rev. (2018) 48:11–20. doi: 10.1016/j.arr.2018.09.002, PMID: 30254001

[52] Phillips CM Shivappa N Hébert JR Perry IJ. Dietary inflammatory index and biomarkers of lipoprotein metabolism, inflammation and glucose homeostasis in adults. Nutrients. (2018) 10:1033. doi: 10.3390/nu10081033, PMID: 30096775 PMC6115860

[53] Langsted A Kamstrup PR Nordestgaard BG. High lipoprotein (a) and high risk of mortality. Eur Heart J. (2019) 40:2760–70. doi: 10.1093/eurheartj/ehy902, PMID: 30608559

[54] Stoll JR Vaidya TS Mori S Dusza SW Lacouture ME Markova A. Association of interleukin-6 and tumor necrosis factor-alpha with mortality in hospitalized patients with cancer. J Am Acad Dermatol. (2021) 84:273–82. doi: 10.1016/j.jaad.2020.03.010 32171811 PMC7486231

[55] O'Sullivan TA Hafekost K Mitrou F Lawrence D. Food sources of saturated fat and the association with mortality: a meta-analysis. Am J Public Health. (2013) 103:e31–42. doi: 10.2105/AJPH.2013.301492, PMID: 23865702 PMC3966685

[56] Bordoni A Danesi F Dardevet D Dupont D Fernandez AS Gille D. Dairy products and inflammation: a review of the clinical evidence. Crit Rev Food Sci Nutr. (2017) 57:2497–525. doi: 10.1080/10408398.2014.967385, PMID: 26287637

[57] Griffiths K Aggarwal BB Singh RB Buttar HS Wilson D De Meester F. Food antioxidants and their anti-inflammatory properties: a potential role in cardiovascular diseases and cancer prevention. Diseases. (2016) 4:28. doi: 10.3390/diseases4030028, PMID: 28933408 PMC5456284

[58] Kennedy ET Ohls J Carlson S Fleming K. The healthy eating index: design and applications. J Am Diet Assoc. (1995) 95:1103–8. doi: 10.1016/S0002-8223(95)00300-2 7560680

[59] Miller PE Lazarus P Lesko SM Muscat JE Harper G Cross AJ. Diet index-based and empirically derived dietary patterns are associated with colorectal cancer risk. J Nutr. (2010) 140:1267–73. doi: 10.3945/jn.110.121780, PMID: 20444952 PMC3499942

[60] Panagiotakos DB Pitsavos C Stefanadis C. Dietary patterns: a Mediterranean diet score and its relation to clinical and biological markers of cardiovascular disease risk. Nutr Metab Cardiovasc Dis. (2006) 16:559–68. doi: 10.1016/j.numecd.2005.08.006, PMID: 17126772

[61] Hu FB. Dietary pattern analysis: a new direction in nutritional epidemiology. Curr Opin Lipidol. (2002) 13:3–9. doi: 10.1097/00041433-200202000-00002, PMID: 11790957

[62] National Cancer Institute, NIH. Accounting for measurement error in dietary intake data. (2011). Available at: http://appliedresearch.cancer.gov/measurementerror (Accessed October 1, 2023).

[63] Satija A Yu E Willett WC Hu FB. Understanding nutritional epidemiology and its role in policy. Adv Nutr. (2015) 6:5–18. doi: 10.3945/an.114.007492, PMID: 25593140 PMC4288279

