# Supplementary material for: Analysis of dietary inflammatory potential and mortality in cancer survivors using NHANES data
Source: Front Nutr. 2024 Sep 13;11:1467259. doi: 10.3389/fnut.2024.1467259 (PMC11427406; doi:10.3389/fnut.2024.1467259)
Supplement: Supplementary file 1 [file Data_Sheet_1.pdf]

Supplementary Materials:

Table S1: The components of DII in the US National Health and Nutrition Examination Survey (NHANES); Figure S1: The sequence of steps in creating the dietary inflammatory index (DII); Figure S2: Dose-response association of DII with the risk of all-cause mortality and cancer mortality among cancer population in the US National Health and Nutrition Examination Survey (NHANES); Figure S3: Stratified analysis of associations of the dietary inflammatory index (DII) with all-cause and cancer mortality among cancer population in the US National Health and Nutrition Examination Survey (NHANES); Table S2: Nutrient components and intake across tertiles of the Dietary Inflammatory Index (DII) score in the US National Health and Nutrition Examination Survey (NHANES)； Table S3: Associations of the dietary inflammatory index (DII) with all-cause and cancer mortality among cancer population in the US National Health and Nutrition Examination Survey (NHANES) in sensitivity analysis.

Supplementary Table 1. The components of DII score in the US National Health and Nutrition Examination Survey (NHANES).

Components	NAHNES (n = 27)
**Pro-inflammatory food parameters**	
Carbohydrate	√
Cholesterol	√
Energy	√
Iron	√
Trans fat	
Protein	√
Vitamin B12	√
Total fat	√
Saturated fat	√
**Anti-inflammatory food parameters**	
Alcohol	√
Monounsaturated fatty acids	√
Onion	
Green/black tea	
Anthocyanidins	
Pepper	
Garlic	
Flavan-3-ol	
Flavones	
Flavonols	
Flavonones	
Isoflavones	
Vitamin A	√
Vitamin C	√
Vitamin D	
Riboflavin	√
Thiamin	√

Niacin	√
Vitamin E	√
β-Carotene	√
Magnesium	√
Selenium	√
Caffeine	√
Fibre	√
Folic acid	√
Polyunsaturated fatty acids	√
Vitamin B6	√
Zinc	√
Eugenol	
Ginger	
n-3 Fatty acids	√
n-6 Fatty acids	√

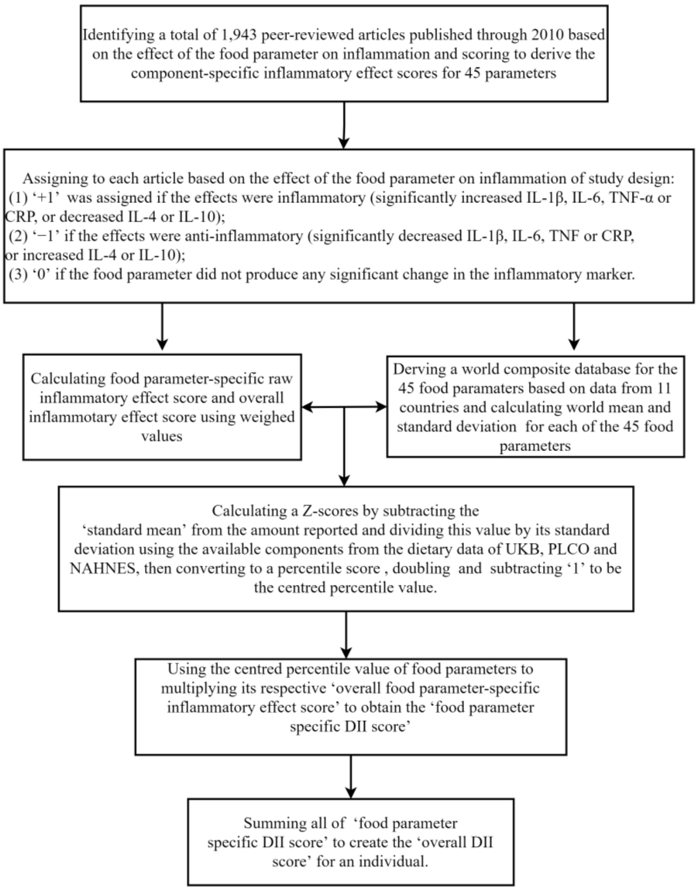

Supplementary Figure 1. The sequence of steps in creating the dietary inflammatory index (DII).

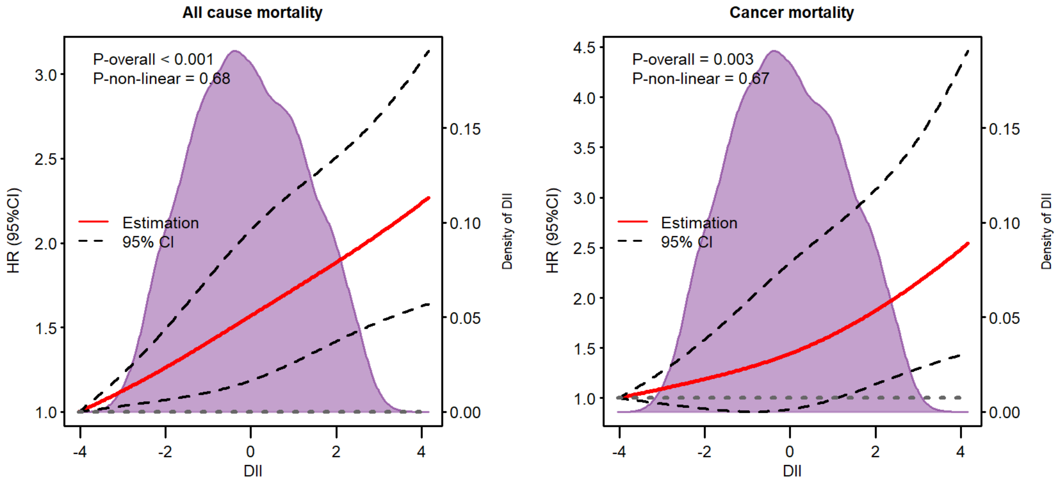

Supplementary Figure 2. Dose-response association of DII with the risk of all-cause mortality and cancer mortality among cancer population in the US National Health and Nutrition Examination Survey (NHANES). Solid splines represented estimated hazard ratios, and cloud areas represented the corresponding 95% CI. The multivariable-adjusted model was adjusted for age, sex, tertiles of energy intake, years from cancer diagnosis to baseline, PIR, marital status ,educational level, race/ethnicity, baseline BMI group, smoking status, and history of comorbidities.

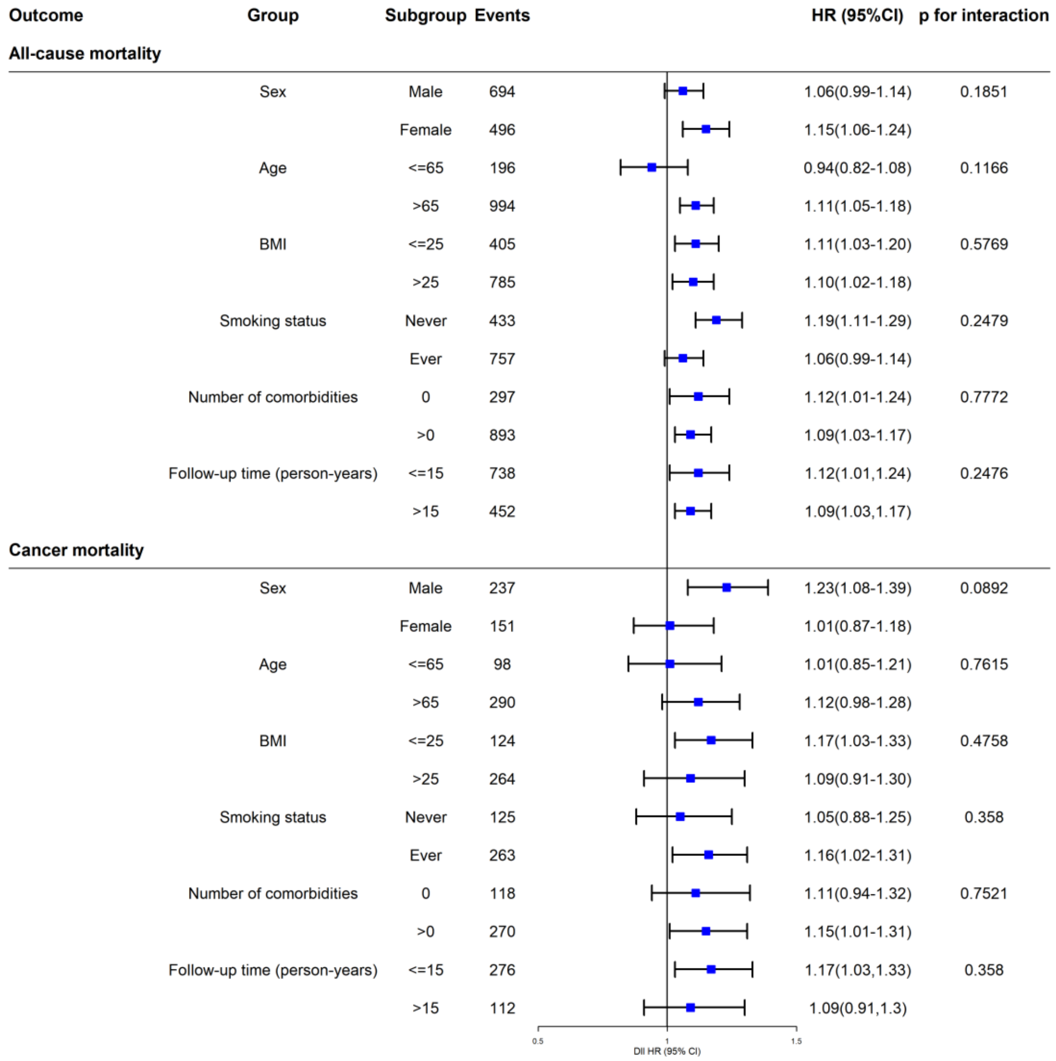

Supplementary Figure 3. Stratified analysis of associations of the dietary inflammatory index (DII) with all-cause and cancer mortality among cancer population in the US National Health and Nutrition Examination Survey (NHANES). Adjusted for age, sex, tertiles of energy intake, years from cancer diagnosis to baseline, PIR, marital status ,educational level, race/ethnicity, baseline BMI group, smoking status and history of comorbidities except for the stratified factor.

Supplementary Table 2. Nutrient components and intake across tertiles of the Dietary Inflammatory Index (DII) score in the US National Health and Nutrition Examination Survey (NHANES) [1].

Components	Tertile 1	Tertile 2	Tertile 3
	-2.07 (0.79)	-0.05 (0.52)	2.11 (0.84)
Alcohol	8.90 (17.76)	5.56 (14.03)	5.72 (24.80)
Vitamin B12	7.46 (12.69)	5.13 (6.13)	3.39 (3.22)

Components	Tertile 1<br>-2.07 (0.79)	Tertile 2<br>-0.05 (0.52)	Tertile 3<br>2.11 (0.84)
Vitamin B6	2.73 (1.07)	1.84 (0.69)	1.16 (0.48)
Caffeine	177.82 (172.92)	169.32 (188.43)	156.03 (182.55)
Carbohydrate	281.05 (84.19)	229.49 (71.69)	179.12 (67.70)
Cholesterol	304.25 (195.62)	275.10 (180.37)	217.85 (146.56)
Energy	2304.70 (612.91)	1874.03 (517.23)	1449.93 (476.88)
Total fat	88.34 (33.23)	72.76 (29.44)	55.14 (23.20)
Fiber	23.98 (8.68)	15.46 (4.97)	9.64 (3.85)
Folic acid	250.61 (182.95)	172.17 (119.10)	113.47 (78.17)
Iron	20.56 (9.30)	14.56 (5.46)	9.83 (3.83)
Magnesium	386.67 (117.79)	268.99 (66.09)	182.77 (56.88)
Monounsaturated fatty acids	32.21 (13.42)	26.02 (11.26)	19.99 (9.05)
Niacin	29.94 (10.37)	21.78 (7.58)	15.30 (5.60)
Protein	92.52 (27.82)	72.92 (22.81)	53.91 (19.91)
Polyunsaturated fatty acids	21.41 (8.90)	16.30 (7.84)	10.98 (5.32)
Riboflavin	2.70 (0.98)	2.07 (0.80)	1.47 (0.59)
Saturated fat	27.03 (12.33)	24.00 (11.14)	19.07 (9.28)
Selenium	126.32 (50.46)	100.42 (37.31)	73.72 (28.88)
Thiamin	2.06 (0.77)	1.52 (0.50)	1.07 (0.39)
Vitamin A	1935.61 (1897.08)	1116.30 (750.49)	595.98 (473.93)
Vitamin C	131.65 (96.10)	84.81 (64.38)	48.50 (45.15)
Vitamin E	11.61 (6.65)	7.09 (3.04)	4.23 (1.93)
Zinc	14.77 (6.80)	10.75 (5.96)	7.44 (3.56)
n-3 Fatty acids	2.20 (1.09)	1.63 (0.85)	1.07 (0.58)
n-6 Fatty acids	18.98 (8.09)	14.48 (7.05)	9.76 (4.76)
β-Carotene	4703.38 (9036.78)	2049.53 (2211.79)	872.47 (1229.28)

[1] Values were mean ± standard error for continuous variables.

Supplementary Table 3. Associations of the dietary inflammatory index (DII) with all-cause and cancer mortality among cancer population in the US National Health and Nutrition Examination Survey (NHANES) in sensitivity analysis [1]

	Tertile 1	Tertile 2	Tertile 2	*P* trend	Per 1-unit DII increment
**Excluded cases with less than 1 year from cancer diagnosis to dietary data gathering**					
All-cause mortality					
No. of deaths	281	341	359	981	
Multivariable-adjusted HR (95% CI)	1.00 (reference)	1.16(0.92-1.46)	1.33(1.03-1.72)	0.0312	1.10(1.04-1.16)
Cancer mortality					
No. of deaths	97	97	118	312	
Multivariable-adjusted HR (95% CI)	1.00 (reference)	1.24(0.79-1.94)	1.26(0.81-1.96)	0.2822	1.12(1.01-1.25)
**Without adjusting BMI**					
All-cause mortality					
No. of deaths	346	416	431	1193	
Multivariable-adjusted HR (95% CI)	1.00 (reference)	1.17(0.95-1.43)	1.31(1.06-1.63)	0.0144	1.09(1.04-1.15)
Cancer mortality					
No. of deaths	117	124	147	388	
Multivariable-adjusted HR (95% CI)	1.00 (reference)	1.25(0.85-1.85)	1.31(0.87-1.97)	0.1872	1.13(1.01-1.25)

[1] All estimates accounted for complex survey designs in NHANES. The multivariable model was adjusted for age, sex, tertiles of energy intake and years from cancer diagnosis to baseline, PIR, marital status ,educational level, race/ethnicity, baseline BMI group, smoking status, and history of comorbidities.
